# Dietary Nitrate Bioactivation at the Diet–Microbiota–Host Interface: The Enterosalivary Cycle, Food Matrix, Microbial Determinants and Health Implications—A Narrative Review Supported by a Structured Literature Search

**DOI:** 10.3390/nu18172841

**Published:** 2026-08-29

**Authors:** Gilda-Diana Buzatu, Ana-Maria Dodocioiu, Eleonora Daniela Ciupeanu-Călugaru, Dumitru Radulescu, Emil-Tiberius Trască

**Affiliations:** 1 Department of Biology and Environmental Engineering, Faculty of Horticulture, University of Craiova, 13 A.I. Cuza Street, 200585 Craiova, Romania; gilda.buzatu@edu.ucv.ro (G.-D.B.); ciupeanudaniela@gmail.com (E.D.C.-C.); 2 Department of Horticulture & Food Science, Faculty of Horticulture, University of Craiova, 13 A.I. Cuza Street, 200585 Craiova, Romania; 3 Department of Surgery, University of Medicine and Pharmacy of Craiova, 2 Petru Rareș Street, 200349 Craiova, Romania; dr_radulescu_dumitru@yahoo.com (D.R.); etrasca@yahoo.com (E.-T.T.)

**Keywords:** dietary nitrate, nitrite, nitric oxide, oral microbiota, gut microbiota, enterosalivary cycle, food matrix, cardiometabolic health

## Abstract

**Background/Objectives:** Dietary nitrate, long framed through food-safety concerns about N-nitroso compound formation, is now also recognised as a substrate of the nitrate–nitrite–nitric oxide pathway. This review aims to define the mechanistic, dietary and host conditions under which nitrate bioactivation becomes functionally relevant, with particular attention to its microbial determinants and to the level of inference the evidence actually supports. **Methods:** We conducted a narrative review supported by a structured literature search (PubMed, Scopus and Web of Science; 1 January 1976 to 14 February 2026; full-text, peer-reviewed, English-language, human-relevant sources; 148 sources retained, of which 93 contributed to the evidence synthesis), with narrative synthesis of mechanistic, interventional, observational and regulatory sources addressing dietary source and food matrix, enterosalivary metabolism, oral and gut microbial function, and health-related outcomes. A PRISMA-style flow diagram summarises the documented screening and inclusion process, and the complete database-specific search strategies are provided in Supplementary Table S1; no meta-analysis was performed because of substantial heterogeneity in designs and outcomes. **Results:** Within the canonical enterosalivary pathway, nitrate-to-nitrite bioactivation is predominantly microbiota-dependent and downstream conversion is chemically conditional: within the enterosalivary cycle, nitrate-reducing bacteria on the tongue dorsum generate the nitrite required for downstream nitric oxide formation, and its conversion in the stomach depends on pH and on matrix constituents. Dietary source and food matrix therefore govern both the delivered dose and the chemistry that follows, so vegetables, beetroot products, inorganic salts, drinking water and processed meat are not interchangeable exposure models. The oral microbiota is the principal microbial determinant of the response, whereas the gut microbiota acts as a context-dependent modifier of intestinal redox tone, barrier function and microbial ecology, supported by markedly weaker human evidence. Nitrate-rich sources reproducibly raise nitrate and nitrite biomarkers, with variable effects on blood pressure, vascular function and exercise efficiency, limited or inconsistent effects on cognition, cerebral blood flow and metabolic endpoints, and a safety profile whose interpretation depends on food matrix, dose, exposure pattern and host context rather than concentration alone. **Conclusions:** We propose the Source–Matrix–Microbiota–Host (SMMH) framework, in which biological impact depends on the interaction between dietary source and dose, food matrix, microbial nitrate-reducing capacity and host susceptibility, rather than on nitrate dose alone, and in which pathway-level, physiological and clinical evidence are kept explicitly distinct. The evidence base is mechanistically robust for the oral microbiota, considerably less defined for the gut microbiota, and variable at the level of validated clinical endpoints; it does not yet support source-independent guidelines or population-level recommendations.

## 1. Introduction

### 1.1. Dietary Nitrate as a Source- and Context-Dependent Exposure

Inorganic dietary nitrate occupies an ambiguous position in nutrition science. It was traditionally approached toxicologically because of its potential involvement in the endogenous formation of N-nitroso compounds under conditions favouring nitrosation [1,2,3,4], and is now also recognised as a bioactive compound of physiological relevance through the nitrate–nitrite–nitric oxide pathway, particularly when consumed within vegetables and other plant-derived foods [1,2,5]. Vegetables, especially leafy greens and beetroot, are among the main dietary sources of inorganic nitrate [6,7,8]. In processed meat, nitrate and nitrite may contribute, together with haem iron, amines, salt, lipid oxidation products and processing conditions, to reactions associated with N-nitroso compound formation [9,10,11]. By contrast, nitrate-rich vegetables are embedded in a matrix containing vitamin C, polyphenols and other redox-active constituents that may support nitrate–nitrite–nitric oxide bioactivity while limiting conditions favourable to nitrosation [1,2,12,13,14]. The health effects of dietary nitrate are therefore inherently source- and context-dependent and cannot be inferred from chemical identity alone, but should be interpreted according to dietary source, food matrix, dose and exposure context [1,2,5,15].

### 1.2. The Enterosalivary Pathway and Its Microbial Dependence

The physiological relevance of dietary nitrate is largely explained by the nitrate–nitrite–nitric oxide pathway [5,16,17]. After ingestion, nitrate is absorbed in the upper gastrointestinal tract and enters the circulation, where a fraction is actively concentrated by the salivary glands and secreted into saliva [5,16,17,18]. In the oral cavity, commensal nitrate-reducing bacteria, particularly those colonising the posterior tongue, convert salivary nitrate into nitrite, a microbiota-dependent step not efficiently performed by mammalian enzymes under normal physiological conditions [5,17,19]. Swallowed nitrite can then be reduced to nitric oxide in the acidic gastric environment and in peripheral tissues, especially under hypoxic or acidic conditions [5,16,17,20]. This route complements the classical L-arginine–nitric oxide synthase pathway and may become particularly relevant when oxygen-dependent synthase activity is limited or endogenous nitric oxide bioavailability is impaired [5,20,21]. The oral microbiota therefore functions as a primary determinant of bioactivation [5,17,19,22,23], and disruption of this activity, notably through antiseptic mouthwash, can reduce salivary nitrite formation and attenuate downstream nitric oxide-mediated responses, including blood pressure regulation [24,25,26,27]. In parallel, intestinal microbial communities have been implicated in nitrogen oxide metabolism and in processes relevant to mucosal defence, inflammatory signalling, barrier function and microbial ecology [28,29,30,31,32,33], although this evidence remains largely mechanistic and less directly characterised in humans [34,35,36].

### 1.3. Objective of the Review

Biological responses to dietary nitrate vary substantially between individuals, reflecting differences in oral nitrate-reducing capacity and oral microbiome composition, as well as background diet, nitrate source and formulation, gastric conditions, oral hygiene, medication use and baseline cardiometabolic characteristics [23,37,38,39,40]. Previous reviews have described the nitrate–nitrite–nitric oxide pathway, the enterosalivary cycle, the role of oral microbiota, and the cardiovascular or ergogenic effects of nitrate intake [1,5,12,39,41,42]; others have addressed oral and/or gut microbial involvement, including links with sports performance and systemic disease [34,35,37,43,44]; and further reviews have discussed the benefit–risk duality of nitrate and nitrite across vegetables, drinking water and processed meat [2,11,45,46,47]. However, there is a lack of comprehensive reviews that integrate, within a single interpretive framework, (i) dietary source and food-matrix effects, (ii) the differential contributions of oral and intestinal microbial metabolism, and (iii) an explicit stratification of the strength of inference from mechanism to clinical relevance.

This review aims to provide a critical, integrative and context-dependent interpretation of dietary nitrate bioactivation, without framing nitrate as a uniformly beneficial or harmful exposure. It distinguishes itself by:

- Integrating dietary source, food matrix, oral and intestinal microbial metabolism, nitric oxide bioavailability and context-dependent safety within one analytical structure;
- Separating the direct, well-supported role of the oral microbiota in reducing salivary nitrate to nitrite from the indirect, context-dependent and less clearly demonstrated role of the gut microbiota in humans [23,34,35,36];
- Proposing the Source–Matrix–Microbiota–Host (SMMH) framework, presented in Section 7.3, which organises the evidence into four interacting determinant domains and three explicit levels of inference: pathway, physiological and clinical, so that biomarker changes are not read as a clinical benefit.

Throughout, “health implications” refer to mechanistically informed biological, physiological and safety-related relevance, not to established clinical recommendations or causal conclusions at the population level.

## 2. Materials and Methods

### 2.1. Design of the Review

This is a narrative review supported by a structured literature search, designed to synthesise the available evidence on dietary nitrate bioactivation at the diet–microbiota–host interface, with a mechanistic, critical and interpretive orientation rather than an aim to meet all methodological requirements of a systematic or meta-analytic review. The PRISMA-style flow diagram is used only as a reporting aid to make record identification, screening and inclusion transparent; it should not be interpreted as a claim of PRISMA-compliant systematic-review methodology. No prospective protocol registration was undertaken; the absence of registration is acknowledged as a limitation in Section 7.6. No meta-analysis was performed because of substantial heterogeneity across study designs, nitrate sources, exposure durations and outcome definitions.

### 2.2. Information Sources and Timeframe

The search was performed in PubMed, Scopus and Web of Science, covering 1 January 1976 to 14 February 2026, and was supplemented by backward citation screening of the reference lists of pertinent original studies and reviews, and by consulting scientific assessments and reports issued by international or regulatory organisations (EFSA, WHO, IARC). Only full-text, peer-reviewed, English-language sources relevant to human physiology were considered as core evidence. The start of the search window corresponds to the first descriptions of nitrate-dependent nitrite formation in human saliva, which define the mechanistic baseline of the field [18,48].

### 2.3. Search Strategy

Search queries were structured around three predefined conceptual blocks addressing (i) dietary nitrate/nitrite exposure, (ii) oral and gut microbial involvement in nitrate bioactivation, and (iii) nitric oxide-related physiological and health outcomes. In PubMed, controlled vocabulary (MeSH) was combined with free-text terms; Scopus was searched using title, abstract and keywords (TITLE-ABS-KEY); and Web of Science Core Collection was searched using the Topic field. The concepts and terms were derived from the review question and established terminology in the nitrate–nitrite–nitric oxide, microbiome and food-matrix literature and agreed upon by the author team; no formally validated search filter was used.

(“Nitrates”[Mesh] OR “Nitrites”[Mesh] OR “dietary nitrate” OR “inorganic nitrate” OR “beetroot juice”) AND (“Microbiota”[Mesh] OR “Mouth”[Mesh] OR “oral microbiome” OR “nitrate-reducing bacteria” OR “gut microbiota” OR “enterosalivary”) AND (“Nitric Oxide”[Mesh] OR “blood pressure” OR “endothelial function” OR “exercise performance” OR “cerebral blood flow” OR “gastrointestinal” OR “N-nitroso compounds” OR “food matrix”).

To increase transparency and reproducibility, the database-specific implementations used the same predefined conceptual domains with explicit platform-specific field and temporal restrictions. Complete database-specific strings, field settings, temporal limits and verification details are provided in Supplementary Table S1. No study-design filters were applied at the search stage in order to maximise sensitivity; English-language, full-text and human-relevance criteria were applied during eligibility assessment rather than used to narrow the database queries.

### 2.4. Eligibility Criteria

**Inclusion:** Mechanistic and biochemical investigations; in vitro and microbiological studies; preclinical animal studies; human intervention trials; observational studies; food-composition and analytical studies; and systematic reviews or meta-analyses, provided they reported data on nitrate/nitrite exposure, bioactivation, microbial nitrate reduction, nitric oxide-related biomarkers, physiological outcomes, or safety.

**Exclusion:** Records without accessible full text; non-English publications; studies addressing nitric oxide biology without any dietary or microbial nitrate component; and duplicate reports of the same dataset.

Guidelines, consensus statements, risk assessments, editorials, methodological reports and previously published reviews were retained as contextual and background sources, not as core evidence for quantitative statements.

### 2.5. Study Selection Process

Records were exported to Zotero (version 7) and deduplicated. Following deduplication, the documented screening-stage dataset comprised 1202 records. Of these, 872 were excluded following title and abstract screening, leaving 330 reports for full-text assessment. A further 182 reports were excluded at the full-text stage: no relevant dietary or microbial nitrate component (*n* = 64); contextual source without data usable for the main synthesis (*n* = 68); full text unavailable (*n* = 21); non-English publication (*n* = 17); and duplicate report of the same dataset (*n* = 12). This left 148 sources retained and cited. Titles, abstracts and full texts were screened independently by two reviewers, with disagreements resolved by consensus. The 41 additional records comprised items identified through backward citation screening and regulatory or organisational documents (EFSA, WHO and IARC) and were incorporated into the common record set before duplicate removal.

**Figure 1:**
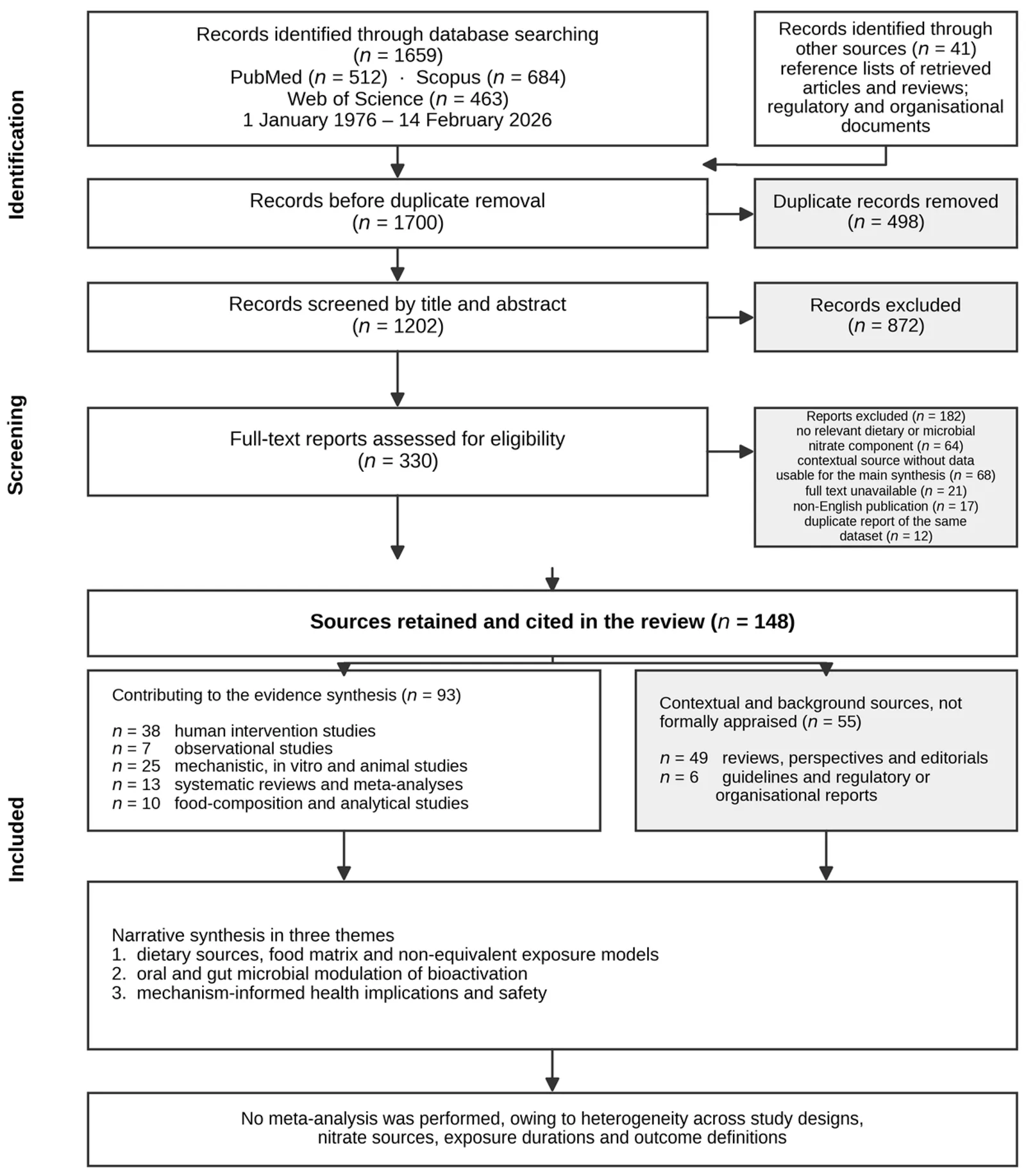
PRISMA-style flow diagram of the documented screening and inclusion process. The diagram begins with the post-deduplication screening set (*n* = 1202). Database-specific search strategies, fields and temporal limits are reported in Supplementary Table S1. Contextual sources, previously published reviews, perspectives, and editorials, together with guidelines and regulatory or organisational documents, were used for background and were not formally appraised, which is why the total number of references exceeds the number of sources contributing to the evidence synthesis.

Sources were retained when they contributed to understanding nitrate exposure, bioactivation, physiological effects, clinical relevance, or safety. Selection was guided by relevance to the conceptual framework rather than by the direction or statistical significance of the reported findings; supportive, inconsistent, neutral and null findings were therefore all retained where they clarified interindividual variability, source- and matrix-dependent effects, or methodological limitations. The documented screening and inclusion process is summarised in Figure 1. Complete database-specific search strategies are reported in Supplementary Table S1.

### 2.6. Study Typology

Of the 148 sources retained and cited, 93 contributed to the evidence synthesis: 38 human intervention studies; 7 observational studies; 25 mechanistic, in vitro and animal studies; 13 systematic reviews and meta-analyses; and 10 food-composition and analytical studies. The remaining 55 were contextual and background sources that were not formally appraised: 49 previously published reviews, perspectives and editorials, and 6 guidelines or regulatory and organisational reports (EFSA, WHO, IARC). This distinction is why the total number of references (*n* = 148) exceeds the number of sources contributing to the evidence synthesis (*n* = 93). The full distribution is shown in Figure 1.

### 2.7. Data Extraction and Synthesis

From each source we extracted design, population and setting, nitrate source and dose, exposure duration, comparator (including the presence of nitrate-depleted controls), microbial and biochemical measures, and reported outcomes. Extraction used a form developed a priori and was cross-checked between reviewers. Because of heterogeneity, a narrative synthesis was performed and organised into three themes: (i) dietary sources, food matrix and exposure models; (ii) oral and gut microbial modulation of bioactivation; and (iii) mechanism-informed health implications and safety.

### 2.8. Quality and Risk of Bias Assessment

Quality appraisal used criteria adapted from the Joanna Briggs Institute checklists and was applied only to the 93 sources contributing to the evidence synthesis; the 55 contextual sources were used for background only and were not formally appraised. Of the 93 appraised sources, 61 raised low concern, 25 moderate concern and 7 high concern. Findings judged to be at a high risk of bias were not used as standalone support for key conclusions.

Strength of inference was interpreted according to study design rather than through a formal grading system. Mechanistic, in vitro and animal studies were used to assess biological plausibility and pathway-level interactions; human intervention studies were considered more directly informative for short-term physiological responses; and observational studies were interpreted cautiously as evidence of associations and dietary patterns rather than causality. Accordingly, three levels of inference were kept distinct throughout: **pathway-level evidence** (absorption, enterosalivary recycling, oral bacterial reduction, salivary nitrite formation, nitric oxide-associated biochemical change); **physiological evidence** (blood pressure, endothelial function, exercise efficiency, cerebral blood flow, gastrointestinal and inflammatory or metabolic parameters); and **clinical or public health relevance** (reproducible effects on meaningful outcomes across diverse populations, longer interventions and realistic dietary contexts).

The qualitative labels used in evidence map are interpretive descriptors rather than formal certainty grades. “Well” or “strongly supported” denotes convergent mechanistic evidence with reproducible human pathway engagement or perturbation; “moderate” denotes multiple human studies with broadly consistent direction but material heterogeneity in magnitude, design or endpoints; and “limited” or “inconsistent” denotes sparse, mixed, null or predominantly indirect/preclinical evidence. Systematic reviews and meta-analyses are used to contextualise consistency and heterogeneity; because no pooled estimate was generated in this review, they are not treated as additional independent datasets on top of their component primary studies.

*Signature statement.* Given explicit eligibility criteria and a transparent selection process, a PRISMA-style flow diagram was used as a reporting aid (Figure 1); this remains a narrative review, and no meta-analysis was performed because of heterogeneity across designs and outcomes.

## 3. Conceptual Framework: The Enterosalivary Nitrate–Nitrite–Nitric Oxide Pathway as the Unifying Mechanism

All three themes that follow are organised around a single mechanistic thread: the best-characterised physiological effects of dietary nitrate depend on its conversion to nitrite and downstream reactive nitrogen species, and each step of that conversion is conditional. Four principles define this thread.

**(i)**  **Enterosalivary nitrate-to-nitrite bioactivation is predominantly microbiota-dependent.** A substantial fraction of circulating nitrate (frequently estimated at up to approximately 25%) is taken up, concentrated and secreted into saliva, where concentrations may exceed those in plasma several-fold [16,17,18,48,49,50]. Because mammalian cells do not efficiently reduce nitrate to nitrite under normal physiological conditions, this conversion depends on the nitrate-reductase activity of commensal bacteria located primarily on the posterior dorsum of the tongue [5,17,18,19]. The quantity of nitrite generated is therefore determined by the abundance, composition and activity of oral nitrate-reducing communities [19,22,23,51,52], which explains why identical doses produce non-identical exposures.
**(ii)**  **Conversion to nitric oxide is chemically conditional.** Nitrite-rich saliva reaching the acidic stomach can be protonated to nitrous acid and decompose into nitric oxide and other reactive nitrogen species, a non-enzymatic reaction favoured by low pH and enhanced by reducing agents such as vitamin C and polyphenols [53,54,55,56]. Gastric nitric oxide may contribute to mucosal blood flow, mucus production, antimicrobial defence and barrier function [33,54,55,56,57,58]. Consequently, elevated gastric pH, acid-suppressive therapy or hypochlorhydria may attenuate this step [35,40,54,55,56], and the same matrix constituents that promote nitric oxide formation also limit nitrosation [14,53,54]. This is the chemical basis for the source-dependence developed in Section 4. Proton pump inhibition provides a clinically relevant example: in a human study, a proton pump inhibitor abolished the blood-pressure-lowering effect of orally ingested nitrite [40]. Because acid suppression may also alter the ecological environment along the upper gastrointestinal tract, its nitrate-specific effect on microbial reducing capacity or physiological responsiveness remains unresolved [34,35].
**(iii)**  **The pathway is complementary, not redundant.** Beyond the stomach, nitrite circulates as a reservoir for nitric oxide generation in blood and tissues, with reduction favoured under hypoxia, ischaemia, or acidosis and mediated by deoxyhaemoglobin, deoxymyoglobin, xanthine oxidoreductase, mitochondrial components and vascular redox systems [20,59,60,61,62,63,64]. The classical L-arginine–nitric oxide synthase route requires oxygen, cofactors and normal enzymatic coupling [21,65]; the nitrate–nitrite–nitric oxide route becomes most relevant precisely where that route is constrained [5,20,59,65]. This asymmetry predicts that responses should be larger in contexts of reduced nitric oxide bioavailability, an expectation that recurs in the cardiovascular, exercise and neurovascular literature (Section 6).
**(iv)**  **The pathway is a network, not a linear sequence.** Its output is shaped simultaneously by dietary source and dose, oral microbiota, salivary secretion, gastric pH, systemic redox state, oxygen availability, tissue metabolism and host health status [5,16,20,23,34,39,54,55,56,59,60]. Timing matters for the same reason: plasma nitrate and nitrite rise after ingestion, whereas salivary measures reflect recycling, so acute effects are typically assessed within a few hours of intake, while chronic intake may involve shifts in oral microbial composition and nitrate-reducing capacity that do not translate proportionally into vascular outcomes [23,38,42,52,66,67,68,69].

Together, these principles imply that dietary nitrate should be treated as a conditional substrate: the relevant biological unit is not the nitrate ion but the combination of source, matrix, microbial capacity and host state. Section 4, Section 5 and Section 6 examine each of these determinants in turn.

## 4. Theme 1: Dietary Sources, Food Matrix, and Non-Equivalent Exposure Models

### 4.1. Overview: What the Evidence Shows

Dietary nitrate intake varies considerably with dietary pattern, vegetable consumption, agricultural practice, season, storage, processing, and drinking-water exposure [6,7,8]. In most diets, vegetables are the principal source, with the highest concentrations in green leafy vegetables such as spinach, lettuce, rocket and chard, and in certain root and stem vegetables including beetroot and celery [6,7,8,70]. Beetroot has received disproportionate attention in human trials because of its nitrate content and its convenience as a standardised source, particularly in studies of blood pressure, endothelial function and exercise [66,67,68,71,72,73,74,75,76,77]. Nitrate content itself varies with species, cultivar, light exposure, cultivation conditions, nitrogen fertilisation, irrigation, maturity, post-harvest storage, processing and culinary preparation [6,7,8,70,78,79]; reduced light, greenhouse or off-season cultivation and intensive fertilisation increase concentrations, whereas greater light exposure, maturation and some preparation methods reduce them or alter the fraction available for ingestion [6,7,8,78,80]. Categories such as “nitrate-rich vegetables” therefore conceal large differences across species, batches, regions and preparation methods. Boiling reduces nitrate through diffusion into cooking water, whereas juices and concentrates deliver higher doses per unit volume than typical whole-vegetable portions [6,78,81,82,83]; fermentation and pickling alter nitrate and nitrite concentrations further [84].

### 4.2. Mechanisms: Why the Matrix Changes the Outcome

The matrix operates through the chemistry set out in Section 3(ii). Nitrate-rich vegetables supply nitrate together with vitamin C, polyphenols, carotenoids, potassium, magnesium, folate and fibre [12,13], constituents that modify redox reactions, nitrite-to-nitric oxide conversion and nitrosation chemistry [12,13,14,53]; vitamin C and polyphenols in particular promote nitric oxide formation from nitrite under acidic gastric conditions [53,54]. In processed meat, nitrite and nitrate are used for curing, colour stability, flavour and microbial control [9,11], but coexist with haem iron, sodium, lipid oxidation products and nitrosatable amines and amides, while smoking or high-temperature treatment favours the formation of compounds with mutagenic potential [9,10,11,85]. The same chemical species therefore carries markedly different implications depending on the matrix in which it is delivered [2,10,11,15,86].

Bioavailability is the second determinant [16,23,48,87]. The biological response to a given dose depends on gastrointestinal absorption, salivary recirculation, oral microbiota composition and nitrate-reductase activity, swallowing of nitrite-rich saliva, gastric pH and subsequent reduction of nitrite in the stomach, blood, and tissues [5,16,17,18,19,20,23,40,48,55,56]. This is why individuals respond differently to identical sources, and why exposure models are not interchangeable: whole vegetables deliver nitrate within a complex plant matrix; isolated nitrate salts isolate the nitrate effect but remove the matrix [86]; and beetroot juice and concentrates occupy an intermediate position, supplying nitrate alongside betalains and polyphenols while differing from habitual intake in processing, concentration and dose standardisation [12,13,53,66,67,68,82,88].

### 4.3. Implications

The findings obtained with nitrate salts or beetroot-based supplements are informative for mechanisms but should not be generalised to all dietary nitrate sources or to vegetable-rich dietary patterns [12,13,53,66,67,68,82]. The distinction also applies over time. Acute intake raises circulating nitrate and nitrite and can influence blood pressure, vascular function, oxygen-use efficiency and exercise outcomes within hours, with variable magnitude [38,66,67,68,72,73,74,75,76,77,89]; longer-term effects depend additionally on sustained intake, oral nitrate-reducing capacity, microbial functional change, baseline cardiovascular risk, medication use and gastric conditions [23,40,69]. Observational studies have reported source-specific associations, vegetable-derived nitrate with lower cardiovascular disease incidence [90] and plant-sourced nitrate and nitrite with lower all-cause, cardiovascular, and cancer mortality [91], but these may reflect both nitrate-related mechanisms and the broader influence of plant-rich dietary patterns and associated lifestyle factors. Table 1 summarises the main exposure contexts, their relevant doses or thresholds, and their distinct safety interpretations.

### 4.4. Open Questions

Isolating nitrate-specific effects from whole-food effects remains the central methodological problem of the field. Nitrate-depleted placebos are useful but do not fully account for interactions among nitrate, betalains, polyphenols and other constituents [74,98]. Direct comparative designs—comparing whole vegetables versus beetroot-derived products versus inorganic salts, against nitrate-depleted controls, with analytically verified doses—are required before source-specific recommendations can be justified.

## 5. Theme 2: Oral and Gut Microbiota as Modulators of Bioactivation

### 5.1. Overview: What the Evidence Shows

The oral cavity, and particularly the posterior dorsum of the tongue, is the main site of nitrate reduction within the enterosalivary cycle; other niches, including dental plaque and mucosal biofilms, contribute to a lesser extent [17,18,19,25,99]. Several genera have been associated with this activity, most consistently *Neisseria* and *Rothia* (Table 2), although nitrate reduction should not be inferred from the presence of individual genera because functional capacity varies across species and strains [19,22,23,37,51,99,100,101]. The strongest functional evidence comes from human experiments using antibacterial or antiseptic mouthwash: suppression of oral bacterial activity reduces nitrate-to-nitrite conversion, lowers salivary and plasma nitrite, and attenuates some nitric oxide-mediated responses, with increased blood pressure or a blunted blood pressure-lowering response reported in several studies [24,25,26,27]. These findings are not arguments against oral hygiene, which remains essential for preventing dental and periodontal disease; the relevant distinction is between targeted oral health practices and broad-spectrum antimicrobial disruption, and formulations differ considerably in potency, active ingredients and effects on specific bacterial groups [25,37,102,103].

By contrast, the contribution of the gut microbiota to dietary nitrate metabolism is less well-established in humans and appears more indirect, secondary and context-dependent [34,35,36]. In vitro and experimental studies indicate that gut bacteria can metabolise nitrate and nitrite through Nar/Nap-associated nitrate reductases and diverse nitrite-reductase pathways, including Nir- and NrfA-associated systems, yielding nitric oxide or ammonium depending on the pathway and organism, with possible effects on local redox balance, microbial competition and ecological stability [28,29,30,104,105], but the magnitude and physiological relevance of these processes in humans remain undefined.

### 5.2. Mechanisms

The tongue biofilm provides reduced oxygen tension, structured anaerobic microenvironments and substrate-rich salivary flow, conditions that support nitrate-reducing activity [17,18,19,25,99]. Net conversion depends on community-level enzymatic activity, gene expression, biofilm architecture, local pH and oxygen gradients, nutrient availability, and interspecies interactions [19,22,23,37,51,52,99,100,101], which explains why oral nitrate-reducing activity varies markedly between individuals at comparable intakes. Repeated nitrate exposure also acts as an ecological pressure: short-term supplementation studies report shifts in nitrate-reducing taxa in profiles considered compatible with oral health and in salivary biochemistry [37,52,100,101], with possible effects on pH and on acidogenic or disease-associated bacteria [37,52,100,101,106]. Direction, magnitude, persistence and clinical relevance depend on baseline microbiota, dose, duration, and design, so nitrate-rich foods should be discussed as potential modulators of oral microbial ecology rather than as established oral health interventions [107]. Periodontal status may modify this capacity further, since periodontal disease alters community composition, inflammation, and biofilm ecology [108]—a plausibly bidirectional relationship that remains a research hypothesis rather than an established pathway.

In the intestine, pH, oxygen availability, nutrient composition, transit time, microbial density and immune interaction differ substantially from the oral cavity, determining whether nitrate and nitrite are reduced to nitric oxide, enter microbial reduction pathways, or support bacterial respiration under low-oxygen conditions [28,29,30,31,32]. Under inflammatory conditions, host-derived nitrate and reactive nitrogen species increase luminal nitrate availability and confer a selective respiratory advantage on facultative anaerobes, particularly *Enterobacteriaceae* such as *Escherichia coli*, whose expansion is discussed in relation to inflammatory dysbiosis and inflammatory bowel disease [31,32,104,109,110]. Critically, this is host-derived nitrate in an inflamed gut, not dietary nitrate in a healthy one; these findings should be read as mechanistic and preclinical, not as evidence that dietary nitrate causes dysbiosis. Nitric oxide itself is dual in the intestine: at physiological concentrations, it supports mucosal blood flow, barrier regulation, mucus production, antimicrobial defence and smooth muscle tone [33], whereas excessive or sustained production, particularly via inducible nitric oxide synthase during inflammation, may promote nitrosative stress, epithelial injury and amplified inflammatory signalling [32,33]. Local intestinal nitrogen oxide chemistry must therefore not be equated with systemic nitric oxide bioavailability derived from the enterosalivary pathway [20,32,33,59,60,65,111,112,113]. Whether high dietary nitrate provides enough additional colonic substrate to alter this competition in active IBD is unknown. The present evidence does not establish that dietary nitrate fuels *Enterobacteriaceae* in IBD because the key mechanistic studies concern host-derived nitrate and experimental inflammatory states [32,109,110]; direct studies should measure luminal nitrate, microbial respiratory activity and inflammatory outcomes in patients.

### 5.3. Implications: Interindividual Variability and Microbiome-Informed Nutrition

Following comparable exposure, some individuals show greater increases in salivary nitrite, plasma nitrite, nitric oxide-related biomarkers or blood pressure responses than others. This variability is partly explained by oral nitrate-reducing capacity and microbiome composition, but also reflects baseline blood pressure, cardiometabolic risk, oral hygiene and mouthwash use, medication exposure, gastric conditions, background diet, age, salivary flow, and physical activity [16,23,26,27,37,38,48,66,67,68,100,101,114]. Age may modulate oral nitrate-reductase activity [115,116], periodontitis may impair reducing capacity [108], and smoking may influence oral nitrate-reducing bacteria and salivary nitrite, although this evidence remains preliminary [117]. Host genetics may also contribute to nitrate recycling. Candidate salivary nitrate-transport mechanisms, including the proposed involvement of sialin/SLC17A5, provide a plausible route for genetic variability [50], but no common human transporter variant has yet been validated as a predictor of salivary nitrate concentration or physiological response within the evidence base reviewed here.

Consequently, “responder” and “non-responder” status should not be treated as a fixed host trait. Oral microbiota composition, nitrate-reducing function, salivary nitrite production and circulating biomarkers are all sensitive to recent intake, background diet, antibacterial mouthwash or antibiotics, oral health, salivary flow, intervention duration, and cardiometabolic context [24,25,26,27,37,100,101,102,114]. Response patterns should therefore be considered dynamic unless confirmed by repeated measurement under standardised conditions over longer follow-up. Salivary nitrate and nitrite measurement, tongue microbiome profiling, and functional nitrate-reduction assays remain promising research tools requiring methodological standardisation and validation against clinically meaningful outcomes [23,37,39,43,101,115,118]. Microbiome-informed nitrate nutrition is, at present, a research concept rather than a validated basis for individualised recommendations [23,34,37,39,101,118].

### 5.4. Open Questions

Three distinctions require resolution. The first is whether functional assays of nitrate-reducing capacity outperform taxonomic composition as predictors of physiological response; the presence of a taxon does not establish activity, temporal stability or predictive value. The second is whether gut microbial mechanisms contribute measurably to systemic responses after dietary nitrate intake, as opposed to modifying intestinal physiology in parallel. Studies addressing the latter must separate dietary nitrate exposure from host-derived nitrate generated during intestinal inflammation [104,109,110]. The third is whether host genetic variation in salivary nitrate transport or downstream nitrite/NO handling improves prediction beyond microbial and clinical phenotyping; candidate transporter genetics should be treated as exploratory until replicated in human response studies.

## 6. Theme 3: Mechanism-Informed Health Implications and Safety

### 6.1. Overview: What the Evidence Shows, by Domain

The strength and consistency of the evidence differ sharply by outcome, and Table 3 maps each domain to its principal mechanism, reported outcomes, and narrative strength of evidence. Three tiers emerge.

*Well supported.* Operation of the enterosalivary cycle, the role of the oral microbiota in nitrate reduction, and reproducible increases in salivary and plasma nitrate and nitrite after intake of nitrate-rich vegetables, beetroot products or inorganic salts are well supported by evidence [5,16,17,18,19,22,23,25,41,48,66,67,68,88,99,119].

*Moderately supported but heterogeneous.* Blood pressure and vascular function: several intervention studies and meta-analyses report reductions, more consistently in systolic than diastolic pressure, influenced by baseline pressure, age, dose, duration, source, cardiometabolic status, and oral nitrate-reducing capacity [23,24,25,26,27,38,53,66,67,68,71,87,89,101,119,120,121,122,123,124,125,126,127,137]. Exercise performance: reductions in the oxygen cost of submaximal exercise and improvements in tolerance and efficiency are reported, more consistently in recreationally active than in highly trained individuals, and vary with dose, timing, duration, exercise type and training status [42,72,73,74,75,76,77]. Instructive counter-examples exist within this tier. Du Toit et al. [101] showed that supplementation with green leafy vegetables and potassium nitrate modified the oral microbiome and selected salivary biomarkers in individuals with elevated blood pressure—evidence of oral microbiome modulation, not of a consistent antihypertensive effect. Fejes et al. [69] showed that four weeks of increased nitrate intake from beetroot juice altered nitrate metabolism in older adults with treated hypertension without improving vascular function or blood pressure.

*Limited, inconsistent or predominantly indirect.* Cognitive function and cerebral blood flow: a short-term high-nitrate protocol increased regional frontal white-matter perfusion in older adults without changing global perfusion [128], and an acute beetroot juice dose modified the prefrontal haemodynamic response and improved serial-subtraction performance in healthy young adults, though not across all cognitive outcomes [129]; a 13-week trial in overweight and obese older adults found no significant effect on cognition or frontal cerebral blood flow at any dose [131], and the available systematic review and meta-analysis found no convincing evidence of consistent benefit [130]. Metabolic regulation: mechanisms linking nitric oxide to muscle perfusion, mitochondrial respiration, glucose handling and inflammation are plausible and supported preclinically [133,134,135], but human trials are mixed or null: beetroot juice raised plasma nitrate and nitrite in type 2 diabetes without improving blood pressure, macro- or microvascular endothelial function or insulin sensitivity [98], and studies in older adults report limited or non-significant vascular and microvascular effects [138,139]. Gastrointestinal physiology: gastric conversion of swallowed nitrite provides plausibility for effects on mucosal blood flow, mucus, antimicrobial defence and barrier function; Björne et al. [57] showed that nitrite-rich saliva increased gastric mucosal blood flow and mucus thickness, but durable clinical benefit in humans is not established, and the dual nature of nitric oxide in inflammation precludes describing nitrate as uniformly protective in gastrointestinal disease [32,33,53,54,55,56,57,58,113,136]. Gut microbiota as a clinical mediator and microbiome-based personalised nutrition remain weakly supported and predominantly indirect [23,28,29,34,35,37,39,43,104,109].

### 6.2. Mechanisms: Why Responses Vary

Variability arises at each conditional step of Section 3. Upstream, it reflects oral nitrate-reducing capacity: disruption by antibacterial mouthwash reduces conversion and attenuates vascular responses [24,25,26,27], so cardiovascular effects depend not only on ingestion but on an intact enterosalivary cycle. Downstream, it reflects the state of the host: the pathway is predicted to matter most where nitric oxide bioavailability is already reduced, which is consistent with larger blood pressure effects reported at higher baseline pressure [38,66,67,68,71,89,137], with the neurovascular hypothesis that nitrate acts as a modulator of cerebral perfusion rather than a direct cognitive enhancer [128,129,130,131,132], and with the observation that established metabolic disease, oxidative stress, vascular dysfunction and medication use may blunt rather than amplify responsiveness [40,65,69,98,111,112,138,139]. Timing contributes independently, since exercise protocols typically administer nitrate two to three hours before testing to align with absorption, salivary recycling and oral reduction kinetics [42,75,76], and acute and repeated protocols do not produce identical responses [75,76].

Matrix effects are the persistent confounder. Beetroot juice supplies betalains and polyphenols that may influence oxidative stress, inflammation, vascular responses and recovery independently of nitrate [81,88,140]; vegetable-based interventions carry fibre, potassium, magnesium, folate and vitamin C [1,12,13,15,45]. Improvements observed after vegetable-based interventions cannot therefore be attributed exclusively to nitrate, and nitrate-depleted placebos, while useful, do not fully resolve matrix interactions [74,98].

### 6.3. Implications: Source-Dependent Safety

Safety cannot be inferred from bioactivation. Nitrite-induced methaemoglobinaemia under specific exposure conditions and the endogenous formation of N-nitroso compounds remain the principal concerns [3,4,9,11,85,86,96]. Importantly, IARC classified ingested nitrate or nitrite as probably carcinogenic to humans *under conditions that result in endogenous nitrosation*, rather than classifying nitrate from all dietary sources as uniformly carcinogenic [3]. Whether nitrate-derived nitrite contributes predominantly to nitric oxide formation or participates in nitrosation depends on nitrite availability, gastric acidity, amines and amides, haem iron, the oxidative environment, processing conditions, and modifiers such as vitamin C and polyphenols [1,2,9,10,11,15,45,53,54,85].

This produces a graded interpretation across the exposure contexts in Table 1. Processed meat constitutes a distinct context in which nitrite coexists with haem iron, amines, salt and lipid oxidation products, and where curing, smoking or high-temperature cooking favour nitrosation chemistry [9,10,11,85]. Vegetable-derived nitrate is delivered with vitamin C, polyphenols, fibre and minerals that shift the same chemistry towards nitric oxide formation [1,2,45,53,54,81,140], a difference that reflects the surrounding matrix, not the nitrate ion itself [1,2,15,45]. This does not imply that vegetable-derived exposure is automatically risk-free, nor that all processed-meat exposures carry identical risk: dose, processing, frequency, duration and population characteristics remain determinative [4,9,10,11,15,45,81,85,86,92,95]. Concentrated products warrant separate consideration; in a short-term human study, concentrated nitrate-rich beetroot juice increased urinary nitrate, nitrite and apparent total N-nitroso compound excretion [94], which demonstrates altered endogenous nitrogen-compound metabolism but does not establish formation of specific carcinogenic N-nitroso compounds or long-term harm.

Vulnerable groups warrant particular attention: infants fed formula prepared with nitrate-contaminated water are especially susceptible to methaemoglobinaemia [86,96]; pregnancy-related concerns apply primarily to contaminated drinking water, where observational evidence suggests possible associations with preterm birth and selected congenital anomalies, though inconsistently across outcomes [86,97]; high habitual processed-meat intake should be interpreted within the broader carcinogenic context of processed meat rather than attributed solely to nitrate or nitrite [10,85]; and unsupervised use of high-dose products by individuals receiving antihypertensive or vasodilatory therapy may warrant clinical caution, although direct evidence of clinically significant interactions is limited [68,71,120,121,122,123]. Regulatory limits and acceptable daily intakes remain essential for risk assessment but do not capture source- and matrix-dependent benefit–risk considerations [4,7,15,45,92,95,96].

### 6.4. Open Questions

Whether biomarker changes ever become sufficient evidence of benefit is the unresolved question of the field. Most controlled studies have assessed nitrate and nitrite biomarkers, blood pressure, vascular function, exercise performance, cerebral blood flow or cognition rather than major cardiovascular events, disease progression or mortality [38,42,81,82,121,123,130,131,135]; observational studies linking vegetable-derived nitrate to cardiovascular outcomes and mortality cannot isolate nitrate from the dietary pattern in which it is embedded [90,91,141]. Long-term safety of repeated exposure to concentrated products likewise remains insufficiently characterised [4,81,94]. Long-term observational data in high vegetable consumers therefore provide indirect reassurance for habitual plant-derived nitrate exposure [90,91,141], but they cannot establish the safety of chronic high-dose supplementation because source, matrix, dose, product composition and residual confounding differ substantially [81,82,92,94,95].

## 7. Discussion

### 7.1. Synthesis of Common Themes

Reading through the unifying framework of Section 3, one finds that the literature converges on a structural finding: the biological effect of dietary nitrate cannot be inferred from the nitrate ion alone. Upstream lie dietary sources and food matrix, which set both the delivered dose and the chemical environment in which nitrite will be resolved, either towards nitric oxide or towards nitrosation. Midstream lies the oral microbiota, the best-characterised and usually dominant nitrate-to-nitrite bioactivation step in the enterosalivary pathway. Downstream lies host state gastric pH, baseline nitric oxide bioavailability, vascular and metabolic status, medication, and age, which determines whether an increase in nitrite availability produces a measurable physiological change. The gut microbiota sits alongside rather than within this sequence, as a context-dependent modifier of intestinal physiology whose contribution to systemic outcomes is not established in humans. Because a substantial portion of the supporting evidence is observational or mechanistic, these relationships should be interpreted as associations and temporal trends rather than confirmed causal effects.

Indirect gut-microbiota effects may also arise through diet–microbiota interactions and microbial metabolites relevant to systemic inflammation and cardiometabolic signalling, including bile-acid and TMAO-related pathways [142,143,144,145,146]. In the SMMH framework, these processes are treated as interactions between the Microbiota and Host domains rather than as a fifth determinant domain: the four domains classify determinants of transportability, whereas these pathways are downstream or parallel mediators. Their nitrate-specific causal contribution remains unproven.

### 7.2. Critical Comparative Analysis

The most informative divergence in the literature is between exposure models rather than between populations. Studies using concentrated beetroot products and inorganic salts converge on reproducible pathway engagement and frequently report short-term physiological effects; studies using whole vegetables and habitual dietary approaches are fewer, longer and less consistent [15,81,82,119,147,148]. This divergence is often read as an inconsistency, but it is better read as a difference in what is being tested: the former isolates the nitrate stimulus at a supraphysiological standardisation, the latter tests a dietary pattern in which nitrate is one constituent among many. A second divergence separates populations in whom nitric oxide bioavailability is presumed low, older adults and hypertensive or cardiometabolically impaired individuals, where mechanistic reasoning predicts larger effects, but several trials report null findings [69,98,131,138,139]. These null results are not anomalies; they delimit the conditions under which pathway engagement fails to translate, and they argue against the assumption that impairment automatically confers responsiveness. A third divergence, between oral and intestinal microbial evidence, is one of evidentiary maturity rather than of biological disagreement: human functional experiments exist for the former and are largely absent for the latter.

Prior syntheses have separately emphasised source and matrix [1,2,12,15,45], the enterosalivary pathway and oral microbiota [5,37,39,41], or translational limitations of supplementation studies [82]. The proposed contribution of the SMMH framework is not a new biochemical pathway, but an explicit cross-study transportability rule that requires these determinants to be considered jointly and separates pathway engagement from physiological and clinical inference.

### 7.3. A Conceptual Proposal: The Source–Matrix–Microbiota–Host (SMMH) Framework

**Figure 2:**
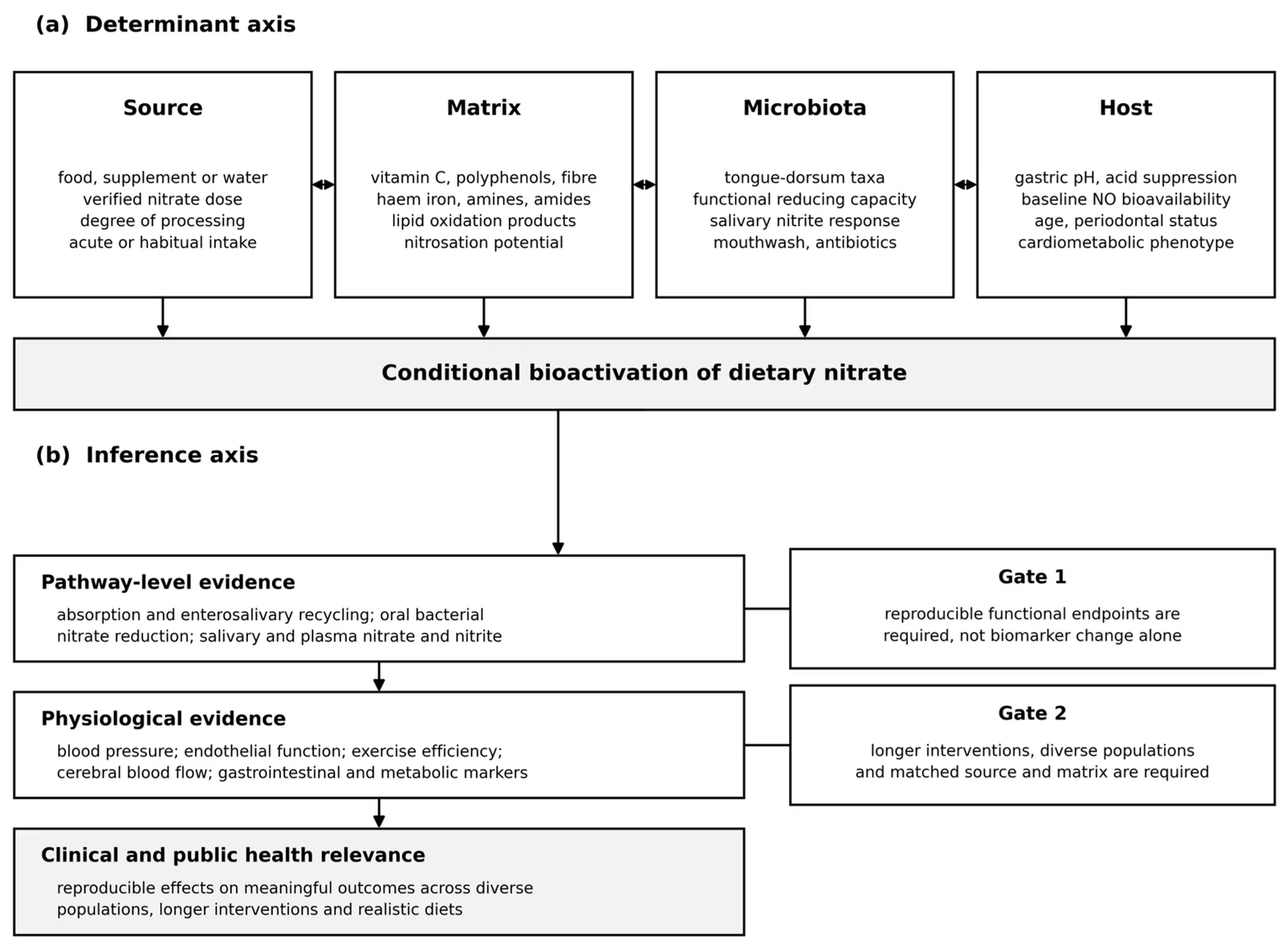
The Source–Matrix–Microbiota–Host (SMMH) framework. (**a**) The determinant axis comprises four interacting domains, each with measurable example indicators, which together govern the conditional bioactivation of dietary nitrate; transportability between studies is expected to be strongest when the relevant domains are matched. (**b**) The inference axis stratifies any claim into pathway-level, physiological, and clinical or public health evidence; the framework proposes that claims should advance only when the evidence specified at each gate is available. NO, nitric oxide. Bidirectional arrows show interactions among determinant domains; one-way arrows show progression through bioactivation and inference levels; horizontal connectors link evidence levels to gates. Gray shading is for visual hierarchy only and has no quantitative meaning.

We propose the SMMH framework (Figure 2) as an organising structure for evaluating dietary nitrate. It has two axes. The **determinant axis** comprises four interacting domains, each with measurable example indicators: **Source** (food versus supplement versus water; analytically verified nitrate dose; degree of processing); **Matrix** (vitamin C, polyphenols, fibre, haem iron, amines/amides, lipid oxidation products; nitrosation-modifying capacity); **Microbiota** (tongue-dorsum nitrate-reducing taxa and, more importantly, functional reducing capacity; salivary nitrite response; mouthwash and antibiotic exposure); and **Host** (gastric pH and acid-suppressive therapy, baseline nitric oxide bioavailability and blood pressure, age, periodontal status, cardiometabolic phenotype, medication). The **inference axis** stratifies any claim into pathway-level, physiological, or clinical/public health evidence, as defined in Section 2.8.

The framework’s operational rule is that a finding may be transported across the determinant axis only when the relevant domains are matched, and may be elevated along the inference axis only when reproducible functional or clinical endpoints, not biomarkers, support it. This makes several common errors explicit: extrapolating from beetroot concentrate to leafy vegetables violates Source and Matrix matching; treating “responder” status as a fixed trait violates the dynamic nature of the Microbiota domain; equating a rise in plasma nitrite with cardiovascular benefit violates the inference axis; and transferring processed-meat risk to vegetable nitrate, or vegetable-nitrate benefit to supplements, violates Matrix matching in both directions. Framed this way, some apparent inconsistencies in the literature may reflect a mismatch across determinant domains or inference levels rather than replication failure alone.

As a research tool, the SMMH framework is intended first as a structured set of covariates rather than a validated numerical score. Trials can pre-specify measures in each domain—source/dose and processing; matrix composition; functional oral nitrate reduction; and host factors such as gastric-pH-modifying medication and baseline phenotype—and test their incremental predictive value for defined outcomes. A composite “SMMH score” is conceivable, but weights, interactions, calibration, discrimination and external validation would need to be derived prospectively; equal weighting should not be assumed. Until such validation exists, the SMMH framework should guide study design and judgments about transportability rather than clinical decision-making.

### 7.4. Knowledge Gaps

Five gaps follow directly from the synthesis and are detailed with recommended approaches in Table 4: (i) the determinants of responder and non-responder phenotypes, which are unlikely to reduce to oral nitrate-reducing capacity alone [23,25,37,39,40,101,108,115,116,148]; (ii) the near-absence of long-term whole-food interventions, since most trials are acute or short-term and use beetroot products or salts [81,82,147,148]; (iii) the unresolved contribution of the gut microbiota in humans, and its separation from host-derived nitrate in inflammation [28,29,30,31,32,33,34,35,36,104,109,110,136]; (iv) safety research that addresses matrix and nitrosation potential rather than nitrate quantity alone, including validated biomarkers for concentrated and repeated exposures [1,2,9,10,11,15,46,81,85,86,94,96]; and (v) the absence of methodological standardisation, without which cross-study comparison will remain unreliable.

### 7.5. Future Directions

Priority designs are comparative and longer. Trials should place nitrate-rich whole vegetables, beetroot-derived products, isolated nitrate salts and nitrate-depleted controls in direct or complementary comparison over intervention periods long enough to test durability, and should report analytically verified nitrate dose, source and formulation, food-matrix characteristics, degree of processing, timing, background diet, and adherence [15,74,81,82,98,147,148]. They should also characterise the microbial and host domains prospectively: salivary nitrate and nitrite dynamics, functional oral nitrate-reducing capacity rather than taxonomy alone, oral hygiene and mouthwash use, recent antibiotic exposure, periodontal status, gastric-pH-modifying medication, age, and cardiometabolic phenotype in the same designs as the clinical endpoints [23,37,39,40,101,108,115,116]. Predictive models built on these variables require prospective validation in independent populations and demonstration of clinically meaningful benefit before microbiome-informed nitrate strategies can inform individualised recommendations. Responder models should also consider candidate host determinants of salivary nitrate transport as exploratory variables, but genotype-based stratification should not be proposed until replicated human associations exist [50]. SMMH-based prediction should be developed and externally validated against pre-specified physiological or clinical endpoints before any composite score is used in practice.

### 7.6. Limitations

This review has several limitations. It was designed as a narrative review with a mechanistic and interpretive focus; although a structured search, explicit eligibility criteria and a PRISMA-style flow diagram were used as reporting aids, it was not intended to meet all methodological requirements of a systematic review, including prospective protocol registration and quantitative synthesis. Selection and interpretation may therefore be affected by reviewer judgement, and relevant studies may have been missed. The evidence base is methodologically heterogeneous: mechanistic studies, short-term human interventions, observational research, systematic reviews, animal models and in vitro experiments. Direct comparison among these sources is limited by differences in nitrate source and formulation, dose, duration, matrix, participant characteristics, oral hygiene, medication use and outcome assessment; for this reason, no meta-analysis or pooled estimate was attempted. Restriction to English-language, full-text sources may have underrepresented relevant findings, and publication bias and selective reporting may affect the available literature, particularly among small interventions assessing numerous physiological or cognitive outcomes; these biases could not be formally assessed within a narrative design. Several mechanisms discussed, particularly those involving the gut microbiota, intestinal inflammation, microbial nitrate respiration and local redox signalling, derive predominantly from experimental, in vitro or preclinical work and provide plausibility rather than evidence of clinically meaningful effects in humans. Finally, observational associations between vegetable-derived nitrate intake and health outcomes cannot establish nitrate-specific causality independently of the broader dietary pattern and food matrix. The conclusions should accordingly be read as a critical, context-dependent synthesis rather than as an assessment of clinical efficacy or a basis for individualised nutritional recommendations.

## 8. Conclusions

Dietary nitrate is best understood as a dietary substrate whose best-characterised enterosalivary bioactivation is strongly microbiota-dependent and context-specific, rather than as a source-independent clinical intervention or a uniform hazard. The strongest evidence concerns the enterosalivary nitrate–nitrite–nitric oxide pathway and the role of oral nitrate-reducing bacteria in generating salivary nitrite [5,17,18,19,22,25,41,99]; human physiological responses are more conditional and variable than this mechanistic framework alone would suggest, and the contribution of the gut microbiota remains a plausible modifier rather than a demonstrated mediator of clinical benefit. The SMMH framework proposed here formalises this conditionality by requiring that findings be matched across source, matrix, microbiota and host before transport, and be elevated from pathway to physiological to clinical claims only on reproducible functional endpoints. Priorities for future work are longer whole-food comparative interventions with nitrate-depleted controls, direct measurement of functional oral nitrate-reducing capacity, clear separation of oral from intestinal microbial mechanisms, and validated safety biomarkers for concentrated exposures. Until such evidence accumulates, the clinical and public health relevance of dietary nitrate should be regarded as biologically plausible and potentially important, but only partially established, the operative question being not whether dietary nitrate is beneficial or harmful, but when, where, through which microbial pathway, and within which food matrix it becomes physiologically and clinically relevant.

## Figures and Tables

**Table 1:** Main exposure contexts for dietary nitrate and nitrite: relevant doses or thresholds, matrix modifiers, safety concerns, and interpretive implications.

Exposure Context	Relevant Dose or Threshold	Matrix and Modifying Factors	Principal Safety Concern	Interpretive Implication
Green leafy vegetables (spinach, lettuce, rocket, chard, celery)	Highly variable with species, cultivar, season, light, fertilisation, storage and preparation; ADI for nitrate 0–3.7 mg/kg bw/day as nitrate ion [92]	Vitamin C, polyphenols, carotenoids, potassium, magnesium, fibre, and folate; no haem iron [12,13,14,53]	Occasional ADI exceedance at very high intakes; more relevant in infants and young children, under improper storage, or with monotonous diets	Interpreted within a plant-rich pattern favourable to NO bioavailability; not equivalent to processed meat, contaminated water or concentrates [1,2,6,7,8,12,13,15,78,79,80,91,92,93]
Beetroot juice and concentrates	≈5–9 mmol nitrate/day (≈310–560 mg/day) in trials; some protocols use higher or acute pre-exercise doses	Nitrate plus betalains, polyphenols and other beetroot phytochemicals; processing alters the nitrate-to-antioxidant ratio [12,13,53,66,67,68,88]	More concentrated than habitual vegetable intake and may exceed the ADI, which is a chronic population benchmark rather than an acute toxicity threshold; long-term safety of repeated high-dose supplementation remains insufficiently characterised. Observational data from vegetable-rich diets provide only indirect reassurance and are not equivalent to chronic concentrated-product exposure [81,82,90,91,92,94]	Useful as a standardised mechanistic and supplementation model; not equivalent to whole-vegetable intake [66,68,72,73,74,75,81,82,88,92,94,95]
Isolated nitrate salts (sodium, potassium nitrate)	Experimentally defined, often comparable to supplementation trials; ADI as above	Defined dose without the plant matrix; formulation, concentration and duration determine exposure [82]	Isolated exposure without vitamin C, polyphenols or fibre; greater concern at high doses, with repeated use, or where nitrosation is favoured	Isolates nitrate-specific mechanisms; findings should not be extrapolated to vegetable-rich patterns [5,15,16,66,67,82,92,95]
Drinking water	WHO guideline values: 50 mg/L nitrate; 3 mg/L nitrite [96]	Water quality, agricultural contamination, age, gastric conditions, absence of protective plant constituents [86,92,95]	Methaemoglobinaemia, particularly in formula-fed infants; possible chronic-exposure, thyroid and reproductive concerns	A food-safety and environmental-contamination issue; not comparable to moderate vegetable intake [4,86,96,97]
Processed meat (nitrite/nitrate as additives)	Depends on product, regulation and consumption frequency; ADI 0.07 mg/kg bw/day for nitrite; [95] 0–3.7 mg/kg bw/day for nitrate [92]	Haem iron, salt, amines and amides, lipid oxidation products; curing, smoking and high-temperature cooking [9,10,11]	Endogenous formation of N-nitroso compounds under nitrosation-favouring conditions	Requires separate safety interpretation; risk cannot be transferred to vegetable-derived nitrate [2,4,9,10,11,14,15,85,92,95]
Concentrated supplements and nitrate-based pre-workout products	Highly variable between products; may equal or exceed doses used in beetroot or salt trials	Absence of a whole-food matrix; co-formulated active ingredients; variable product composition and nitrate content	Uncertain actual dose, repeated or long-term use, and combination with other actives	Should be discussed separately from dietary vegetables; long-term safety outside controlled settings insufficiently characterised [4,15,44,65,81,82,92,94,95]

ADI, acceptable daily intake; bw, body weight; NO, nitric oxide.

**Table 2:** Oral bacterial genera associated with nitrate reduction and their proposed relevance to enterosalivary nitrate metabolism.

Genus or Group	Reported Physiological Relevance	Ecological Niche	Interpretive Caution
*Neisseria*	Most frequently reported nitrate-reducing taxon in oral microbiome studies [22,23,37,99,100,101]	Tongue dorsum and oral biofilm; high nitrate-reductase activity	Contribution to salivary nitrite depends on community composition, nitrate exposure and host context
*Rothia*	Consistently associated with oral nitrate metabolism [22,23,37,99,100,101]	Tongue dorsum; metabolically active biofilms	Indicator of a nitrate-responsive profile rather than a proven determinant of response
*Veillonella*	Associated with nitrate/nitrite metabolism in some studies [19,22,51,52]	Anaerobic and facultative niches within oral biofilms	Role varies across studies and ecological contexts
*Actinomyces*	Reported nitrate-reducing potential [19,22,51,52,99]	Dental plaque and mucosal biofilms	Contribution depends on biofilm structure, local pH, oxygen gradients and interspecies interactions
*Prevotella*, *Haemophilus*, *Granulicatella*	Additional taxa reported in oral nitrate-reduction studies [22,37,99,100,101]	Variable oral niches	Community-dependent contribution; no uniform single-genus effect

Across all taxa, function should not be inferred from presence alone: nitrate-reducing capacity varies between species and strains and is better captured by functional assays than by taxonomic composition.

**Table 3:** Evidence map: physiological and health-related domains, principal mechanisms, reported outcomes, and narrative strength of evidence.

Domain	Principal Mechanism	Reported or Proposed Outcomes	Narrative Strength of Evidence	Refs
Enterosalivary nitrate–nitrite–NO pathway	Absorption, salivary concentration, oral bacterial reduction, and gastric and tissue NO formation	Pathway engagement demonstrable across compartments, particularly under hypoxic or acidic conditions	Mechanistically well established	[5,16,41,87]
Oral microbiota and nitrate reduction	Bacterial nitrate reductase on the tongue dorsum	Salivary nitrite formation; attenuation after antibacterial mouthwash	Strongly supported	[22,23,25,37,39]
Nitrate/nitrite biomarker response	Substrate delivery and enterosalivary recycling	Reproducible increases in salivary and plasma nitrate and nitrite; magnitude varies with microbiota, dose, source, matrix, gastric pH, medication	Strongly supported	[23,66,67,68,119]
Blood pressure and vascular function	Increased NO bioavailability; sGC activation; smooth-muscle relaxation; endothelial signalling	Reductions in systolic more than diastolic pressure; variable improvements in flow-mediated dilation and arterial stiffness	Moderate to strong for short-term physiological effects; heterogeneous for durable clinical relevance	[65,66,67,68,71,89,90,111,112,120,121,122,123,124,125,126,127]
Exercise performance and muscle function	Improved mitochondrial efficiency; reduced O_2_ cost of ATP production; muscle perfusion and contractile efficiency	Reduced O_2_ cost of submaximal exercise; improved tolerance and time to exhaustion, chiefly in recreationally active individuals	Moderately supported; context-dependent	[42,72,73,74,75,76,77]
Cognition and cerebral blood flow	Neurovascular coupling via nitrite-derived NO under high metabolic demand or reduced NO bioavailability	Region-specific increases in frontal perfusion; inconsistent acute cognitive effects; null findings in longer trials	Limited and heterogeneous	[128,129,130,131,132]
Metabolic regulation and insulin sensitivity	NO interaction with muscle perfusion, mitochondrial respiration, glucose uptake, inflammation	Proposed effects on insulin sensitivity and glucose metabolism; human trials mixed or null	Limited and inconsistent	[34,98,133,134,135]
Gastrointestinal physiology and mucosal defence	Gastric NO from swallowed salivary nitrite	Increased mucosal blood flow and mucus thickness; antimicrobial defence; dual effects in inflammation	Mechanistically plausible; clinically limited	[5,32,33,53,54,55,56,57,113,136]
Gut microbiota as a mediator of clinical effects	Microbial nitrate/nitrite reduction; redox, barrier and ecological modulation	Altered intestinal redox and microbial ecology; *Enterobacteriaceae* respiratory advantage under inflammation	Weak to limited; predominantly indirect and preclinical	[28,29,35,104,109,110]
Oral microbiota and oral health	Nitrate as substrate and ecological pressure within oral biofilms	Shifts towards nitrate-reducing and health-associated profiles; effects on caries or periodontitis not demonstrated	Promising mechanistically; clinically unvalidated	[37,100,101,106,107,114,118]
Safety and nitrosation	Nitrite availability, gastric pH, haem iron, amines/amides, oxidative conditions, antioxidants	N-nitroso compound formation, chiefly in processed-meat and other nitrosation-favouring contexts	Moderately to strongly supported conceptually and toxicologically	[1,3,4,9,10,11,15,81,82,83,84,85,86,92,94,95]
Extrapolation from biomarkers to clinical benefit	-	Biomarker change demonstrates bioactivation, not durable benefit	Limited; requires caution	[38,82,121,130,131]
Microbiome-based personalised nitrate nutrition	Oral profiling, salivary nitrite, functional reducing capacity	Potential responder stratification	Promising but unvalidated	[23,34,35,37,39,43]

NO, nitric oxide; sGC, soluble guanylate cyclase. Categories represent an interpretive narrative assessment based on mechanistic consistency, availability of human intervention and meta-analytic evidence, and extent of translation into physiological or clinical outcomes; they do not constitute a formal certainty-of-evidence or GRADE assessment.

**Table 4:** Knowledge gaps and research priorities for dietary nitrate, the microbiota and nitric oxide bioavailability.

Research Gap	Scientific Rationale	Recommended Approach
Determinants of responder and non-responder phenotypes	Physiological responses vary substantially between individuals, and oral nitrate-reducing capacity alone is unlikely to explain this	Measure salivary nitrate and nitrite dynamics, functional oral nitrate-reducing capacity, oral microbiome profile, periodontal status, gastric-pH-modifying medication, background diet, age, and cardiometabolic status alongside clinical endpoints in the same designs
Long-term whole-food interventions	Most trials are acute or short-term and use concentrates or salts, limiting inference about habitual intake and durability	Compare nitrate-rich whole vegetables, beetroot products, inorganic salts and nitrate-depleted controls over longer periods; report analytically verified dose, matrix, processing, background diet and adherence
Contribution of the gut microbiota	Oral reduction is far better established than intestinal metabolism as a determinant of systemic NO bioavailability	Separate oral-mediated bioactivation from indirect intestinal mechanisms and from host-derived nitrate in inflammation; integrate biomarkers with metagenomic and targeted functional profiling in hypertension, metabolic disease, IBD and ageing
Food matrix and nitrosation potential	Safety interpretation depends on matrix composition as well as dose, duration, and exposure context, not nitrate quantity alone	Compare vegetables, processed meats, contaminated water, salts and concentrates using validated nitrosation and oxidative biomarkers, documenting matrix, processing, duration and gastric conditions
Methodological standardisation	Heterogeneity in exposure assessment, design, microbiome characterisation and outcome measurement prevents cross-study comparison	Standardise reporting of dose and analytical verification, source, formulation, timing, duration, nitrate-depleted controls, oral hygiene and mouthwash use, recent antibiotics, gastric-pH-modifying medication, comorbidities, and outcome methods

IBD, inflammatory bowel disease; NO, nitric oxide.

## Data Availability

No new data were created or analysed in this study. All sources supporting the synthesis are listed in the reference list. Data sharing is not applicable to this article.

## Supplementary Materials

The following supporting information can be downloaded at: https://www.mdpi.com/article/10.3390/nu18172841/s1, Table S1: Database-specific search strategies and verification details.
