# Supplementary material for: Dietary Nitrate Bioactivation at the Diet–Microbiota–Host Interface: The Enterosalivary Cycle, Food Matrix, Microbial Determinants and Health Implications—A Narrative Review Supported by a Structured Literature Search
Source: Nutrients. 2026 Aug 29;18(17):2841. doi: 10.3390/nu18172841 (PMC13567488; doi:10.3390/nu18172841)
Supplement: Supplementary file 1 [file nutrients-18-02841-s001.zip › nutrients-4491673-supplementary.pdf]

Supplementary Material

Dietary Nitrate Bioactivation at the Diet–Microbiota–Host Interface

Table S1. Database-specific search strategies and verification details.

To improve transparency and reproducibility, the three predefined conceptual search blocks were implemented using database-specific syntax and field restrictions. The strategies below were documented and verified on 22 August 2026 using the original review coverage window. They are provided as a reproducible implementation of the predefined search framework rather than as verbatim exports of the original database sessions.

Database	Search field / interface	Exact database-specific search strategy	Temporal limits and verification details
**PubMed**	MeSH terms combined with free-text terms	("Nitrates"[Mesh] OR "Nitrites"[Mesh] OR "dietary nitrate" OR "inorganic nitrate" OR "beetroot juice")<br>AND ("Microbiota"[Mesh] OR "Mouth"[Mesh] OR "oral microbiome" OR "nitrate-reducing bacteria" OR "gut microbiota" OR "enterosalivary")<br>AND ("Nitric Oxide"[Mesh] OR "blood pressure" OR "endothelial function" OR "exercise performance" OR "cerebral blood flow" OR "gastrointestinal" OR "N-nitroso compounds" OR "food matrix")	Original review coverage: 1 January 1976–14 February 2026 (applied as the review-level publication-date coverage criterion). Search verification date: 22 August 2026. No study-design filter.
**Scopus**	Advanced document search; TITLE-ABS-KEY	TITLE-ABS-KEY("nitrates" OR "nitrites" OR "dietary nitrate" OR "inorganic nitrate" OR "beetroot juice")<br>AND TITLE-ABS-KEY("microbiota" OR "mouth" OR "oral microbiome" OR "nitrate-reducing bacteria" OR "gut microbiota" OR "enterosalivary")<br>AND TITLE-ABS-KEY("nitric oxide" OR "blood pressure" OR "endothelial function" OR "exercise performance" OR "cerebral blood flow" OR "gastrointestinal" OR "N-nitroso compounds" OR "food matrix")	Publication-year filter: 1976–2026. Search verification date: 22 August 2026. No document-type, language, subject-area, source-type or open-access filters were applied at the search stage.
**Web of Science Core Collection**	Fielded Search; Topic field (TS); Editions: All	("nitrates" OR "nitrites" OR "dietary nitrate" OR "inorganic nitrate" OR "beetroot juice")<br>AND ("microbiota" OR "mouth" OR "oral microbiome" OR "nitrate-reducing bacteria" OR "gut microbiota" OR "enterosalivary")<br>AND ("nitric oxide" OR "blood pressure" OR "endothelial function" OR "exercise performance" OR "cerebral blood flow" OR "gastrointestinal" OR "N-nitroso compounds" OR "food matrix")	Publication Date: 1 January 1976–14 February 2026. Search verification date: 22 August 2026. No study-design filter.

Notes. The same three conceptual blocks were retained across databases; only the database-specific indexing syntax and field restrictions were harmonised. PubMed retained controlled vocabulary (MeSH) together with free-text terms; Scopus used TITLE-ABS-KEY; Web of Science Core Collection used Topic. The Scopus interface applied the temporal restriction at publication-year level (1976–2026), whereas Web of Science permitted an exact Publication Date interval. English-language, full-text and human-relevance criteria were applied during eligibility assessment and were not used as database-query filters. These specifications are provided to ensure reproducibility of the database-specific implementation of the predefined search framework.
